# A coordinated PCP-Cardiologist Telemedicine Model (PCTM) in China’s community hypertension care: study protocol for a randomized controlled trial

**DOI:** 10.1186/s13063-017-1970-z

**Published:** 2017-05-25

**Authors:** Lei Xu, Wei-Yi Fang, Fu Zhu, Hong-Guang Zhang, Kai Liu

**Affiliations:** 1 Department of Cardiology, Shanghai Chest Hospital, Shanghai JiaoTong University, Shanghai, China; 2 Department of Cardiology, Shanghai XuHui Hospital, Zhongshan Hospital, FuDan University, Shanghai, China; 3 CareLinker Co., Ltd., Shanghai, China

**Keywords:** Randomized controlled trial, Telemedicine, Hypertension, Primary care, Medication adherence

## Abstract

**Background:**

Hypertension is a major risk factor for cardiovascular disease, and its control rate has remained low worldwide. Studies have found that telemonitoring blood pressure (BP) helped control hypertension in randomized controlled trials. However, little is known about its effect in a structured primary care model in which primary care physicians (PCPs) are partnering with cardiology specialists in electronic healthcare data sharing and medical interventions. This study aims to identify the effects of a coordinated PCP-cardiologist model that applies telemedicine tools to facilitate community hypertension control in China.

**Methods/design:**

Patients with hypertension receiving care at four community healthcare centers that are academically affiliated to Shanghai Chest Hospital, Shanghai JiaoTong University are eligible if they have had uncontrolled BP in the previous 3 months and access to mobile Internet. Study subjects are randomly assigned to three interventional groups: (1) usual care; (2) home-based BP telemonitor with embedded Global System for Mobile Communications (GSM) module and unlimited data plan, an app to access personal healthcare record and receive personalized lifestyle coaching contents, and proficiency training of their use; or (3) this plus coordinated PCP-cardiologist care in which PCPs and cardiologists share data via a secure CareLinker website to determine interventional approaches. The primary outcome is mean change in systolic blood pressure over a 12-month period. Secondary outcomes are changes of diastolic blood pressure, HbA1C, blood lipids, and medication adherence measured by the eight-item Morisky Medication Adherence Scale.

**Discussion:**

This study will determine whether a coordinated PCP-Cardiologist Telemedicine Model that incorporates the latest telemedicine technologies will improve hypertension care. Success of the model would help streamline the present community healthcare processes and impact a greater number of patients with uncontrolled hypertension.

**Trial registration:**

ClinicalTrials.gov, NCT02919033. Registered on 23 September 2016.

**Electronic supplementary material:**

The online version of this article (doi:10.1186/s13063-017-1970-z) contains supplementary material, which is available to authorized users.

## Background

Hypertension is one of the major causes of death globally and affects more than 20% of the adult population in China, according to the latest statistics [1]. Multiple lines of evidence have suggested that lowering blood pressure (BP) with anti-hypertensive medications and a more healthful lifestyle would decrease mortality and disability from severe complications [2–4]. However, hypertension remains inadequately treated through routine provider visits and patient self-management. In China, the majority of hypertension care is provided by primary care physicians (PCPs) in community practices. The imbalance between the small number of available PCPs and the vast number of patients has limited the average PCP visit to less than 9 minutes. Hypertension-specific assessments such as reviewing BP trends, ocular exams, and renal function tests are not often performed, and education about medication adherence and behavioral changes may not be provided as necessary.

The advent of mobile healthcare technologies and telemedicine systems has demonstrated tremendous potential to improve the effect of chronic disease management of hypertension, diabetes, cardiac disorders, and respiratory diseases [5–9]. A recent meta-analysis indicated that blood pressure telemonitoring could reduce systolic blood pressure (SBP) by 4.7 mm Hg and diastolic blood pressure (DBP) by 2.5 mm Hg, with an increased hypertension control rate [10, 11]. In diabetic care, a more robust app-based glycemic control algorithm has proven effective in reducing HbA1C by 1.9% in cluster-randomized trials [9, 12–14]. These beneficial effects are considered to result from enhanced disease awareness, self-management, and patient engagement by healthcare professionals [15–18]. It is therefore conceivable that broader implementation of telemedicine systems may help restore the balance of supply and demand in community hypertension care services by mobilizing patient involvement and improving provider’s efficiency.

To combat the growing public health challenge of hypertension, regulators in Shanghai, China are piloting a novel community healthcare PCP-Cardiologist Telemedicine Model (PCTM) in which PCPs are collaborating more closely with cardiologists of larger comprehensive hospitals by leveraging a telemedicine system that integrates remote BP monitoring with healthcare decision support engines. The present paper describes a three-arm randomized controlled trial (RCT) to evaluate the effectiveness of this model in which BP data and personal healthcare information are collected, stored, and displayed by a mobile app in sync with the cloud engine, and personalized healthcare delivery can be provided in a proactive and preventive way by the joint effort of PCPs and cardiologists. By comparing the BP control outcomes of (1) usual care, (2) patient self-management using telemedicine tools, and (3) the PCTM, which includes the coordinated cardiologist services plus the same set of telemedicine tools, we will be able to draw more precise conclusions about this PCTM in hypertension management and determine whether a similar model should be developed for other chronic conditions. Furthermore, as our previous study conducted in more than 3000 retail pharmacies has implicated that improved medication compliance might be the main mechanism underling better BP control, we will perform a Morisky analysis to identify the correlation between medication adherence and the effect of hypertension care in this trial [19, 20].

## Methods/design

### Study design

**Fig. 1:**
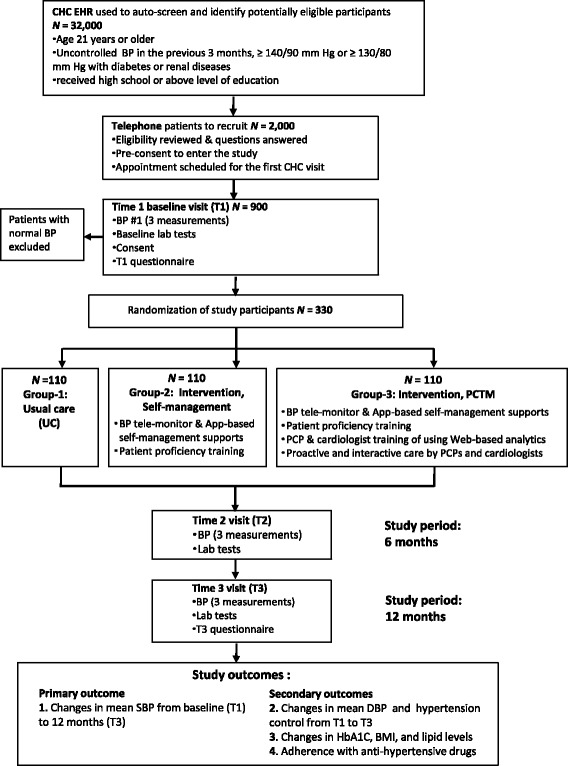
Study design of the coordinated PCP-Cardiologist Telemedicine Model (PCTM)

The coordinated PCTM study is a 12-month, pragmatic three-arm trial aiming to compare two telemedicine-based hypertension interventions with usual care. Group 1 participants receive usual care (UC) that complies with China’s Hypertension Prevention and Treatment Guideline 2010 (the national guideline) [21]. Group 2 patients additionally receive a BP telemedicine system including a BP telemonitor and a mobile app. Group 3 participants receive all of the above plus coordinated services jointly provided by their PCPs and a designated team of cardiologists who have access to the secure CareLinker website and review patient data with PCPs once every 3 months. PCP-directed proactive interventions such as phone consultations, requests for an immediate clinic visit, or referrals may also be given to Group 3 participants if their relevant PCP receives alerts produced by the CareLinker cloud engine as a result of detecting abnormal BP variability. A total of 330 enrolled patients will be randomized to each group in a 1:1:1 allocation ratio, and all outcome measures will be assessed at baseline (T1), 6 months (T2), and 12 months (T3) throughout the study duration (Fig. 1). We hypothesize that timely healthcare information exchange and closer collaboration between PCPs and cardiologists will improve community BP control. The Standard Protocol Items: Recommendations for Interventional Trials (SPIRIT) checklist is presented in Additional file 1 [22].

### Setting

This study will be taking place in the XuHui District in Shanghai, China. The four participating community healthcare centers (CHCs) are classified as tier 1 clinics; each has approximately 30–40 practicing PCPs, and they provide a full range of primary care services to nearly 330,000 residents in total. Cardiologists are mainly recruited from Shanghai Chest Hospital, which is located in the same district and is one of the largest chest specialty hospitals in the nation. Potentially eligible patients are identified and referred by PCPs from CHCs, where all data collection will be conducted including baseline and follow-up tests, except for home-based BP telemonitoring.

### Eligibility: inclusion and exclusion criteria for patients

Inclusion criteria of the trial are as follows: (1) age 21 years or older; (2) a clinical diagnosis of hypertension with uncontrolled BP in the previous 3 months, and currently taking or about to take anti-hypertensive medications; (3) received high school or higher level of education; (4) active user of smart phone (Android or Apple) and mobile apps; (5) the average of three BP measurements during the screening visit at the CHC is ≥140/90 mm Hg, or ≥130/80 mm Hg if the patient has diabetes or renal disease; (6) the ability to give informed consent.

Medical exclusion criteria include the following: acute coronary syndrome, heart failure, cardiac arrhythmia, stroke within the past 3 months, renal failure, cancer, dementia, severe or acute psychiatric illness, pregnancy or intention to be pregnant in the next 18 months, and hospitalization within 3 months. Additional exclusion criteria include participation in another clinical trial, arm circumference >32 cm (this may affect the accuracy of BP measurement due to the cuff size limit of the telemonitor), and unwillingness to comply with the 12 months intervention duration.

### Withdrawal

Withdrawal criteria include the following: (1) the participant intends to drop out of the study; (2) the participant is unwilling to follow the study protocol such as not visiting the clinic as scheduled or having the lab tests performed; (3) the participant is withdrawn due to critical medical reasons. For patients who withdraw from the study, we will obtain a consent form to retrieve data directly from their electronic healthcare records (EHRs) in the CHCs and compare their outcome indicators with those from the patients who complete the study.

### Participant recruitment

We estimate that each participating CHC has at least 8000 registered patients with hypertension who have been receiving the regular community hypertension care (i.e., usual care), resulting in more than 32,000 patients being available for this recruitment. Potential participants are identified either through direct PCP referrals or by screening CHCs’ EHRs following three eligibility criteria: (1) age 21 years or older, (2) had uncontrolled BP in the previous 3 months, i.e., ≥140/90 mm Hg; (3) received high school or higher level of education. Eligible participants are first screened over the phone for interest in participation, experience in smart phone and app use, and willingness to provide the consent form. Those who remain eligible are invited to their registered CHC to attend the screening sessions with at least two PCPs and one research electronic engineer who instructs participants on the use of the BP telemonitor and ensures that participants have the appropriate Android or Apple smart phone to download and use the CareLinker app. If the participant has uncontrolled BP, as defined in the above inclusion criterion 5, during the screening visit and signs the consent, the research nurse will continue the procedure by collecting baseline laboratory tests and study questionnaires. The eligible patients will be randomized to the control or to one of the two intervention groups. The recruitment process is expected to take 12 months to complete from September 2016 to September 2017.

### Outcomes

#### Primary outcome measures

The primary endpoint of the trial is changes in mean SBP from baseline (T1) to 12 months (T3) measured using the BP telemonitor (Bliss BL928). The 12 months BP readings will be determined by taking the average of three BP measurements at the follow-up visit to the CHC. All BP data are collected and uploaded simultaneously to the trial database.

#### Secondary outcomes

The key secondary endpoints are changes in mean DBP and hypertension control rate defined as BP <140/90 mm Hg or <130/80 mm Hg (for patients with diabetes or renal diseases) following the national guidelines [21] and changes in measures related to hypertension complications (HbA1C, body mass index (BMI), and lipid levels) from baseline (T1) to 6 months (T2) and 12 months (T3). The other secondary outcome is anti-hypertensive medication adherence, which is assessed by the self-reported, eight-item Morisky Medication Adherence Scale (MMAS) modified to focus on BP drugs at baseline (T1) and 12 months (T3) [20]. These outcomes are measured for the three study groups and compared between the control and intervention groups.

### Sample size and power calculation

The sample size required for this RCT is 330 patients based on the following assumptions. The RCT is powered to detect a clinically meaningful difference in average change between baseline and 12 months SBP of 7 mm Hg and DBP of 5 mm Hg between Group 1and Group 3. We assume that the standard deviation for difference in SBP from baseline to 12 months is 16.5 mm Hg and the difference in DBP from baseline to 12 months is 11.5 mm Hg. Using a two-sample comparison of the mean change between Group 1 and Group 3, a sample size of 88 patients per group is required to have 80% power at the 95% confidence level. This number is further adjusted to 110 per group to account for an estimated dropout rate of 20% during the 12 months follow-up. To reduce this potential dropout, we will provide all participants an additional 2 years of free health consultation services from PCPs and cardiologists as an incentive after they complete the trial. All analyses will be performed according to the intention-to-treat (ITT) principle.

### Randomization process

We use a block randomization design to ensure balance within CHCs and baseline SBP measurements. We set each CHC as a block, and patients are randomized to three groups in each block with a rate of 1:1:1. A study statistician is solely responsible for generating the randomized allocation sequence using computer software (MATLAB version 2014a) and assigning patients to the intervention or control groups. The allocation sequence is concealed in opaque envelopes from all study researchers until the interventions are assigned. Furthermore, an engineer from CareLinker who does not know the grouping information is in charge of training participants of Groups 2 and 3 to use the BP telemonitor and app in order to ensure an equal level of training between the two intervention groups. Due to the nature of the intervention, the study is an open-label trial to all participating patients, physicians, and coordinators at each site.

### Group 1: usual care

After enrollment into the study, the UC group (Group 1) patients are managed by their PCPs at the registered CHCs as usual. The healthcare services offered to them comply with the national guideline and include designated follow-ups by PCPs once every 1–3 months for stage 1 (≥140/90 mm Hg) hypertension patients, once every 1 month for stage 2 (≥160/100 mm Hg), and once every 2 weeks for stage 3 (≥180/110 mm Hg) patients [21]. The standard basic measures include blood pressure, fasting blood glucose, serum blood lipids, electrocardiogram (EKG), renal function, weight, and BMI. Patients receiving UC are encouraged to use a logbook to record home BP measurements and present the results during the CHC visit. In addition to medication adjustment or prescription refill, PCPs will review BP trend according to the logbook records or history data in the EHR, provide lifestyle coaching such as dietary, weight management, and physical activity advice, prescribe necessary lab tests, and make referrals to specialists for further treatment of hypertension or comorbidities. PCPs are also responsible for promoting national and regional hypertension management guidelines during each patient encounter. Similar to the intervention groups, UC patients will have three study visits, at baseline (T1), 6 months (T2), and 12 months (T3), to complete study tests and questionnaires.

### Group 2: Intervention, self-management using the telemedicine tool

Patients randomly assigned to this group receive a BP telemedicine system developed by CareLinker Inc. (Shanghai, China) to facilitate BP self-management in addition to all the usual care components. A more detailed description has been reported previously [23]. In brief, the system consists of (1) a BP telemonitor with embedded GSM module that can upload BP readings to the cloud database immediately after the self-measurement; (2) a mobile app that allows patients to manually input healthcare data, display history BP measurements and lab test results, receive personalized lifestyle coaching contents and medication reminders, and communicate with PCPs about hypertension-related health concerns through text messaging.

### Group 3: Intervention, managed in the PCP-Cardiologist Telemedicine Model (PCTM)

Group 3 participants receive all the interventions described above for Groups 1 and 2. In addition, PCPs and cardiologists are provided access to the secure CareLinker website where they can review patient healthcare data including all BP measurements, lab test results, medications in use, and comorbidities if any. They can also use the auto analytics tools of the website to view the BP average, BP trend, and the risk score of each patient developing cardiovascular disease (CVD). The CVD risk scoring was developed according to the published data [24, 25]. Text message alerts of abnormal BP variability detected by the cloud engine will be pushed to the PCPs’ app when they occur, and proactive interventions including phone consultations, medication dosage adjustments, or referrals can be offered to patients. A joint case review session will be set once every 1–2 months for PCPs and partnering cardiologists to exam patients’ disease progresses to determine if further medical treatments need to be administered. The proprietary web-based analytic module also produces automated medication recommendations to PCPs based on each patient’s disease condition.

All patients in Groups 2 and 3 receive proficiency training in using the BP telemonitor and mobile app. PCPs and cardiologists in Group 3 receive training on access and use of the password-protected CareLinker website.

### Data collection and management

The processes of data collection, storage, analysis, display, and delivery are achieved through the interplay of the web server, web application software, General Packet Radio Service (GPRS)-enabled BP telemonitor, and user-end applications (apps) for patients or physicians. All these components are deployed on computer servers owned by and located within CareLinker and operated behind the company firewall.

Patients’ blood pressures are self-measured using the BP telemonitor (Bliss, Shenzhen China), and the readings are encrypted with the GEA4 algorithm and auto-sent to the web server through the mobile network GPRS protocol. The GEA4 is a widely used encryption algorithm published by the European Telecommunications Standards Institute (ETSI). Each BP telemonitor has its unique device ID bound to one patient ID, the change of which is subject only to the clinical trial administrator in the case of device failure. Auto-submitted BP measures by the BP telemonitor help avoid errors or bias that may otherwise occur in the manual input process. Lab tests of HbA1C and blood lipids are performed at baseline and at each follow-up visit, and the blood samples will not be stored. The test results and other medical information are manually recorded into the patient app by research nurses, and error-checked with format, range, and logic rules built into the app before being sent to the web server. An additional designated research staff will proofread all patients’ health information by comparing with the original reports. The study procedure and data collection are detailed in the SPIRIT figure (Additional file 2).

The user-end apps use Hypertext Transfer Protocol over Secure Socket Layer (HTTPS) to communicate data with the web server. At the sender side, the checksum of the data is calculated by the Message-Digest Algorithm (MD5), encrypted by the Data Encryption Standard (DES) algorithm, and sent out through HTTPS. At the receiver side, the received data are decrypted and their checksum is calculated. Only when the checksum of the received data equals to the received checksum, indicating that the data are not modified during the communication and the integrity is verified, are the received data accepted by the receiver. The HTTPS, MD5, and DES are commonly used communication protocols or encryption algorithms for Internet security. Both apps can be operated on mobile devices running Android 3.0/+ or iOS 7.0/+.

The open-source Java Servlet Container Apache Tomcat 8.0 (Apache Software Foundation, Forest Hill, MD, USA) is used as the web server, and the web application software is developed on the base of Spring Framework, which is an open-source application framework and inversion of control container for the Java platform. The database uses MySQL 5.7, an open-source relational database management system (RDBMS). Two instances of MySQL configured as master and slave are running simultaneously. The data are automatically synchronized from the master to the slave, and the slave can be auto-switched to perform as the master if the original master fails. Regular backups are carried out to prevent accidental data loss. All patients, PCPs, and cardiologists have their unique IDs, and their login passwords are encrypted and kept anonymous to the database administrator. The database is also password protected, with access only to the administrator and staff members whose IPs have been previously registered in a white list. The research staffs is only provided with read permission to the database.

### Study management

A steering committee has been established to serve as the main governing body of this study. It is composed of cardiologists and clinical research scientists from Shanghai Chest Hospital and epidemiologists from Shanghai JiaoTong University, School of Medicine. The major responsibility of the steering committee is to evaluate, advise on, and approve the study protocol with modifications, communicate with the Ethics Committee, and supervise the practice of community hypertension care in accordance with China’s national hypertension management guideline.

The sponsor of the project is Shanghai Chest Hospital, which is indemnified for any harms as the result of trial participation. A project management team consisting of the principal investigator and the clinical trial operation director has been formed to manage the everyday operation of the study. The operation director is responsible for all decisions on the trial management and intervention delivery.

An independent, external monitor will carry out onsite monitoring, overseen by the study sponsor, once every month at each site following the risk-based monitoring plan established for the study. Safety reporting will follow the safety guidelines of the Project Monitoring Plan, and all adverse events will be recorded in the database.

An Independent Data Monitoring and Safety Committee, consisting of physicians and bioethicists from Shanghai Chest Hospital and statisticians from Shanghai JiaoTong University, School of Medicine, has been established for the trial. This committee is independent from the sponsor and has no competing interests. The committee will be in charge of auditing the trial once every quarter and will meet every 6 months to oversee all ethical and safety issues of the study. The Committee Charter is available from the operation director who manages and coordinates the trial.

### Statistical hypothesis, methods, and analyses

The formal null hypothesis to be tested in this study is that there is no difference in mean change in SBP over a 12-month period among patients from the intervention and control groups. If a significant difference is found among the three groups, we will perform pairwise comparisons, i.e., Group 1 vs Group 2, Group 1 vs Group 3, and Group 2 vs Group 3. As our main goal is to examine whether the most intensive care (Group 3, the PCTM intervention) is more effective in BP control than usual care (Group 1), the alternative hypothesis in this comparison is that Group 3 shows a significant improvement of BP from baseline to 12 months follow-up.

We will conduct the primary statistical analysis on an ITT basis with full endeavor to acquire a complete dataset of every patient. The collected trial data will be coded and entered into MATLAB version 2014a and SPSS version 22 for statistical analysis. Comparative statistics of means or percentage rates of different study arms will be presented with two-sided 95% confidence intervals (CIs), and statistical tests will be reported as a two-sided significance level of 5%. A fully specified Statistical Analysis Plan (SAP) that includes detailed analytics of the trial datasets will be prepared and completed before locking the database.

Baseline data will be analyzed and presented using numerical summaries and graphical illustrations. We will perform *F* tests for continuous variables and Pearson’s chi-square tests for categorical variables to assess the difference of baseline characteristics among the three groups. To analyze the primary outcome, we will use a linear mixed model to identify the effectiveness while adjusting the baseline using the following covariates as appropriate: baseline SBP, age, gender, BMI, education levels, exercise, drinking patterns, tobacco use, and CHCs. Only those covariates found to be statistically significant to the outcomes will be selected as explanatory variables for inclusion in the final model. All statistical tests are performed using *F* tests. We intend to use Fisher’s protected least significant difference (LSD) method to protect against multiple comparisons, followed by pairwise comparisons between study groups only if the overall *F* test is significant. The secondary endpoints of DBP, HbA1C, blood lipid levels, and medication adherence will be determined separately between baseline and the follow-up time points for patients of the three study arms. The analytical approach will be identical to those described in the above section for primary outcome analysis.

Missing data will be assumed to be missing at random; therefore, the linear mixed model has already accounted for this assumption. We will use multiple imputation methods to analyze the missing data.

We will also carry out subgroup analyses of the primary and secondary outcomes stratified by the following characteristics at baseline: BMI (<24, ≥24), hypertension stage (stage1, stage 2, and stage 3), education level (non-college, college, or above), presence of one or more comorbidities, and self-reported medication adherence. No formal interim analysis of the primary and secondary outcomes is planned, but progress reports will be prepared and presented to PCPs and partnering cardiologists every 3 months.

### Adverse events reporting

Serious adverse events will be self-reported at the 6 months (T2) and 12 months (T3) visits to CHC. These events include hospitalization longer than 24 hours, emergency room visits, and other potentially relevant adverse cases of death, acute cerebro-cardiovascular accident, and kidney failure. Research staff members will review the reporting and patient’s healthcare record to determine the correlation to the intervention and trial participation.

## Discussion

China is experiencing an epidemic of chronic diseases, among which hypertension and its complications top the list [1, 26–29]. The traditional model of physician-centered clinic visit has proven ineffective, as a very small number of available PCPs is facing an ever-growing number of patients, leading to limited time to be spent on each patient for collecting information and making exams and therapeutic decisions. New initiatives aiming to improve the efficiency of care delivery such as adopting EHRs, promoting community healthcare guidelines, and using mobile healthcare technologies have been put into action, yet in China few RCTs have been conducted to validate their effectiveness [30]. The other challenge the community healthcare service is confronting is that PCPs may not have sufficient experience to identify and manage hypertension-relevant CVD complications in time. To fulfill the task of improving population BP control to contain CVD risks, the healthcare regulators in Shanghai, China are testing a novel hypertension management model — PCTM — in which PCPs are closely partnering with cardiologists from university hospitals in data sharing and disease treatment by taking advantage of a set of telemedicine tools that allow home-based telemonitoring, app-based lifestyle coaching and text consultation, and auto risk analysis to assist proactive intervention [31]. The present randomized trial is thus designed to provide evidence for future integration into the existing healthcare systems.

Trials from other countries have examined the effects of BP or diabetic control in various primary care setups, and most of these studies found improved outcome measures after interventions. In general, three types of interventional models can be differentiated. The first is patient-centered care delivery. This approach has been trialed to strengthen disease awareness and medication adherence, and recent studies of such often involve adoption of tele-healthcare tools such as an app, text messaging, or telemonitoring devices [32–39]. The second is provider-centered care delivery. The focus of this model is on physician support, with the assistance of nurses, clinical pharmacists, medical educators, dietitians, or clinical decision support algorithms [40–45]. The third and latest approach is centered on the combination of both models [46–51]. Meta-analyses of hypertension care studies and the results from a four-arm diabetic care study all lead to the conclusion that provider-assisted self-management is more effective in healthcare outcomes [9, 11, 52–55]. In contrast to these trials that recruited medical assistants such as nurses as PCP support for intervention, the current PCTM study has the objective to instead add specialists from secondary or tertiary medical centers to intensify the care process. This model is particularly designed to fit into the currently revamping scheme of China’s community healthcare system to support the hypothesis that extending the role of specialists and making them more available to community healthcare services can enhance their effectiveness.

Besides testing the efficacy of the intervention, this study will have several other impacts if successful. First of all, this model can be replicated to apply to the management of other chronic conditions, as the care of diabetes, cardiac disease, and asthma/chronic obstructive pulmonary disease follows similar clinical pathways of telemonitoring, lifestyle, and medical interventions. Next, it provides the feasibility and acceptability evidence of employing mobile technologies to deliver healthcare services by capitalizing on the world’s largest population pool of smart phone users. The upward trend of elderly people using mobile apps for social networking in large cities in China has prepared them with the skills and proficiency to receive clinical care through the mobile platform. Policy makers in other geographic regions across the country could therefore develop and roll out similar telemedicine services. Last but not the least, surveys led by various health bureaus all indicate that patients in China have the perception of receiving better quality of care if specialists from tertiary medical centers are members of their healthcare team. Therefore, the model of integrating specialist service may enhance patient adherence to coaching and medication and may eventually translate to medical outcomes.

We also acknowledge limitations in this study. First, this is an open-label trial, as both patients and physicians are aware of the grouping information. Patient bias, especially for those in the control group, may occur when they are not compliant with follow-up clinic visits and even choose to discontinue the participation, while physician bias may occur when they provide interventions to the test group patients. We attempt to address this issue through intensive training of participating physicians and research nurses prior to the beginning of this study, and by setting up a project management team to continuously oversee the study progress to ensure that the protocol is being properly followed. Second, the recruited patients may reflect only a subpopulation who are frequent users of smart phones and mobile apps. Their demographic and healthcare profile may be distinctive from the rest of the hypertensive population, who are of different ages, at different geographic locations, and have less access to the Internet. Finally, the present study only involves four CHCs and one university hospital in Shanghai; thus, the findings and the PCTM model under study may not be generalized to other regions in China.

### Trial status

This trial is ongoing. The study began in September 2016, and follow-up is expected to be completed by March 2018 (Additional file 2).

## Additional files

Additional file 1: The SPIRIT checklist. (PDF 366 kb)

Additional file 2: SPIRIT figure. (PDF 311 kb)

## Abbreviations

CHC: Community healthcare center
HbA1C: Glycosylated hemoglobin
PCP: Primary care physician
PCTM: PCP-Cardiologist Telemedicine Model
RCT: Randomized controlled trial.

## References

[1] Yang G, Wang Y, Zeng Y, Gao GF, Liang X, Zhou M (2013). Rapid health transition in China, 1990-2010: findings from the Global Burden of Disease Study 2010. Lancet.

[2] Law MR, Morris JK, Wald NJ (2009). Use of blood pressure lowering drugs in the prevention of cardiovascular disease: meta-analysis of 147 randomised trials in the context of expectations from prospective epidemiological studies. BMJ..

[3] Dickinson HO, Mason JM, Nicolson DJ, Campbell F, Beyer FR, Cook JV (2006). Lifestyle interventions to reduce raised blood pressure: a systematic review of randomized controlled trials. J Hypertens.

[4] Sundstrom J, Arima H, Woodward M, Jackson R, Karmali K, Lloyd-Jones D (2014). Blood pressure-lowering treatment based on cardiovascular risk: a meta-analysis of individual patient data. Lancet.

[5] Kumar N, Khunger M, Gupta A, Garg N (2015). A content analysis of smartphone-based applications for hypertension management. J Am Soc Hypertens.

[6] Quinn CC, Clough SS, Minor JM, Lender D, Okafor MC, Gruber-Baldini A (2008). WellDoc mobile diabetes management randomized controlled trial: change in clinical and behavioral outcomes and patient and physician satisfaction. Diabetes Technol Ther.

[7] Pfaeffli Dale L, Whittaker R, Jiang Y, Stewart R, Rolleston A, Maddison R (2015). Text message and Internet support for coronary heart disease self-management: results From the Text4Heart randomized controlled trial. J Med Internet Res.

[8] Fernandez-Granero MA, Sanchez-Morillo D, Leon-Jimenez A (2015). Computerised analysis of telemonitored respiratory sounds for predicting acute exacerbations of COPD. Sensors (Basel).

[9] Quinn CC, Shardell MD, Terrin ML, Barr EA, Ballew SH, Gruber-Baldini AL (2011). Cluster-randomized trial of a mobile phone personalized behavioral intervention for blood glucose control. Diabetes Care.

[10] Omboni S, Caserini M, Coronetti C (2016). Telemedicine and m-health in hypertension management: technologies, applications and clinical evidence. High Blood Press Cardiovasc Prev.

[11] Omboni S, Gazzola T, Carabelli G, Parati G (2013). Clinical usefulness and cost effectiveness of home blood pressure telemonitoring: meta-analysis of randomized controlled studies. J Hypertens.

[12] Quinn CC, Gruber-Baldini AL, Shardell M, Weed K, Clough SS, Peeples M (2009). Mobile diabetes intervention study: testing a personalized treatment/behavioral communication intervention for blood glucose control. Contemp Clin Trials.

[13] Quinn CC, Sareh PL, Shardell ML, Terrin ML, Barr EA, Gruber-Baldini AL (2014). Mobile diabetes intervention for glycemic control: impact on physician prescribing. J Diabetes Sci Technol.

[14] Quinn CC, Shardell MD, Terrin ML, Barr EA, Park D, Shaikh F (2016). Mobile diabetes intervention for glycemic control in 45- to 64-year-old persons with type 2 diabetes. J Appl Gerontol.

[15] Margolis KL, Asche SE, Bergdall AR, Dehmer SP, Maciosek MV, Nyboer RA (2015). A successful multifaceted trial to improve hypertension control in primary care: why did it work?. J Gen Intern Med.

[16] O'Connor PJ, Schmittdiel JA, Pathak RD, Harris RI, Newton KM, Ohnsorg KA (2014). Randomized trial of telephone outreach to improve medication adherence and metabolic control in adults with diabetes. Diabetes Care.

[17] Weltermann B, Viehmann A, Kersting C (2015). Hypertension management in primary care: study protocol for a cluster randomized controlled trial. Trials..

[18] Heisler M, Hofer TP, Klamerus ML, Schmittdiel J, Selby J, Hogan MM (2010). Study protocol: the Adherence and Intensification of Medications (AIM) study—a cluster randomized controlled effectiveness study. Trials..

[19] Xu L, Zhang Y, Chen Y, Li YJ, Chen D, Wang T (2016). The role of pharmacist-led hypertension management in China’s community medical care service: a retrospective analysis of real-world big-data samples. Precision Med Cardiol.

[20] Oliveira-Filho AD, Barreto-Filho JA, Neves SJ, Lyra Junior DP (2012). Association between the 8-item Morisky Medication Adherence Scale (MMAS-8) and blood pressure control. Arq Bras Cardiol.

[21] Liu LS (2011). 2010 Chinese guidelines for the management of hypertension. Zhonghua Xin Xue Guan Bing Za Zhi.

[22] Chan AW, Tetzlaff JM, Gotzsche PC, Altman DG, Mann H, Berlin JA (2013). SPIRIT 2013 explanation and elaboration: guidance for protocols of clinical trials. BMJ..

[23] Zhang HG, Liu K, Kong WJ, Tian F, Yang YR, Feng C, et al. A mobile health solution for chronic disease managment at retail pharmacy. 2016 IEEE 18th International Conference on e-Health Networking, Applications and Services (Healthcom). 2016. p.1-5. doi:10.1109/HealthCom.2016.7749455.

[24] Wu Y, Liu X, Li X, Li Y, Zhao L, Chen Z (2006). Estimation of 10-year risk of fatal and nonfatal ischemic cardiovascular diseases in Chinese adults. Circulation.

[25] Collaborative Group (2003). A study on evaluation of the risk of ischemic cardiovascular diseases in Chinese and the development of simplified tools for the evaluation. Chin J Cardiol.

[26] Li LM, Rao KQ, Kong LZ, Yao CH, Xiang HD, Zhai FY (2005). A description on the Chinese national nutrition and health survey in 2002. Zhonghua Liu Xing Bing Xue Za Zhi.

[27] Sheng CS, Liu M, Kang YY, Wei FF, Zhang L, Li GL (2013). Prevalence, awareness, treatment and control of hypertension in elderly Chinese. Hypertens Res.

[28] Wang L, Kong L, Wu F, Bai Y, Burton R (2005). Preventing chronic diseases in China. Lancet.

[29] Yang G, Kong L, Zhao W, Wan X, Zhai Y, Chen LC (2008). Emergence of chronic non-communicable diseases in China. Lancet.

[30] Hallberg I, Taft C, Ranerup A, Bengtsson U, Hoffmann M, Hofer S (2014). Phases in development of an interactive mobile phone-based system to support self-management of hypertension. Integr Blood Press Control..

[31] Glynn LG, Hayes PS, Casey M, Glynn F, Alvarez-Iglesias A, Newell J (2013). SMART MOVE — a smartphone-based intervention to promote physical activity in primary care: study protocol for a randomized controlled trial. Trials..

[32] Zullig LL, Melnyk SD, Goldstein K, Shaw RJ, Bosworth HB (2013). The role of home blood pressure telemonitoring in managing hypertensive populations. Curr Hypertens Rep.

[33] Kirwan M, Vandelanotte C, Fenning A, Duncan MJ (2013). Diabetes self-management smartphone application for adults with type 1 diabetes: randomized controlled trial. J Med Internet Res.

[34] Logan AG, Irvine MJ, McIsaac WJ, Tisler A, Rossos PG, Easty A (2012). Effect of home blood pressure telemonitoring with self-care support on uncontrolled systolic hypertension in diabetics. Hypertension.

[35] Stoddart A, Hanley J, Wild S, Pagliari C, Paterson M, Lewis S, et al. Telemonitoring-based service redesign for the management of uncontrolled hypertension (HITS): cost and cost-effectiveness analysis of a randomised controlled trial. BMJ Open. 2013;3(5). doi: 10.1136/bmjopen-2013-002681. 10.1136/bmjopen-2013-002681 PMC3657667 23793650

[36] Thiboutot J, Sciamanna CN, Falkner B, Kephart DK, Stuckey HL, Adelman AM (2013). Effects of a web-based patient activation intervention to overcome clinical inertia on blood pressure control: cluster randomized controlled trial. J Med Internet Res.

[37] Kim JY, Wineinger NE, Steinhubl SR (2016). The influence of wireless self-monitoring program on the relationship between patient activation and health behaviors, medication adherence, and blood pressure levels in hypertensive patients: a substudy of a randomized controlled trial. J Med Internet Res.

[38] Hosseininasab M, Jahangard-Rafsanjani Z, Mohagheghi A, Sarayani A, Rashidian A, Javadi M (2014). Self-monitoring of blood pressure for improving adherence to antihypertensive medicines and blood pressure control: a randomized controlled trial. Am J Hypertens.

[39] McManus RJ, Mant J, Bray EP, Holder R, Jones MI, Greenfield S (2010). Telemonitoring and self-management in the control of hypertension (TASMINH2): a randomised controlled trial. Lancet.

[40] Hirsch JD, Steers N, Adler DS, Kuo GM, Morello CM, Lang M (2014). Primary care-based, pharmacist-physician collaborative medication-therapy management of hypertension: a randomized, pragmatic trial. Clin Ther.

[41] Carter BL, Ardery G, Dawson JD, James PA, Bergus GR, Doucette WR (2009). Physician and pharmacist collaboration to improve blood pressure control. Arch Intern Med.

[42] Carter BL, Coffey CS, Ardery G, Uribe L, Ecklund D, James P (2015). Cluster-randomized trial of a physician/pharmacist collaborative model to improve blood pressure control. Circ Cardiovasc Qual Outcomes.

[43] O'Neill JL, Cunningham TL, Wiitala WL, Bartley EP (2014). Collaborative hypertension case management by registered nurses and clinical pharmacy specialists within the Patient Aligned Care Teams (PACT) model. J Gen Intern Med..

[44] Hunt JS, Siemienczuk J, Pape G, Rozenfeld Y, MacKay J, LeBlanc BH (2008). A randomized controlled trial of team-based care: impact of physician-pharmacist collaboration on uncontrolled hypertension. J Gen Intern Med.

[45] Qudah B, Albsoul-Younes A, Alawa E, Mehyar N (2016). Role of clinical pharmacist in the management of blood pressure in dialysis patients. Int J Clin Pharm.

[46] Green BB, Cook AJ, Ralston JD, Fishman PA, Catz SL, Carlson J (2008). Effectiveness of home blood pressure monitoring, Web communication, and pharmacist care on hypertension control: a randomized controlled trial. JAMA.

[47] Magid DJ, Olson KL, Billups SJ, Wagner NM, Lyons EE, Kroner BA (2013). A pharmacist-led, American Heart Association Heart360 Web-enabled home blood pressure monitoring program. Circ Cardiovasc Qual Outcomes.

[48] Green BB, Anderson ML, Ralston JD, Catz SL, Cook AJ (2013). Blood pressure 1 year after completion of web-based pharmacist care. JAMA Intern Med.

[49] Margolis KL, Asche SE, Bergdall AR, Dehmer SP, Groen SE, Kadrmas HM (2013). Effect of home blood pressure telemonitoring and pharmacist management on blood pressure control: a cluster randomized clinical trial. JAMA.

[50] Omboni S, Sala E (2015). The pharmacist and the management of arterial hypertension: the role of blood pressure monitoring and telemonitoring. Expert Rev Cardiovasc Ther.

[51] Lee P, Liu JC, Hsieh MH, Hao WR, Tseng YT, Liu SH (2016). Cloud-based BP system integrated with CPOE improves self-management of the hypertensive patients: a randomized controlled trial. Comput Methods Programs Biomed..

[52] Omboni S, Guarda A (2011). Impact of home blood pressure telemonitoring and blood pressure control: a meta-analysis of randomized controlled studies. Am J Hypertens.

[53] van Vugt M, de Wit M, Cleijne WH, Snoek FJ (2013). Use of behavioral change techniques in web-based self-management programs for type 2 diabetes patients: systematic review. J Med Internet Res.

[54] AbuDagga A, Resnick HE, Alwan M (2010). Impact of blood pressure telemonitoring on hypertension outcomes: a literature review. Telemed J E Health.

[55] Santschi V, Chiolero A, Colosimo AL, Platt RW, Taffe P, Burnier M (2014). Improving blood pressure control through pharmacist interventions: a meta-analysis of randomized controlled trials. J Am Heart Assoc.

