# Supplementary material for: A coordinated PCP-Cardiologist Telemedicine Model (PCTM) in China’s community hypertension care: study protocol for a randomized controlled trial
Source: Trials. 2017 May 25;18:236. doi: 10.1186/s13063-017-1970-z (PMC5445306; doi:10.1186/s13063-017-1970-z)
Supplement: Supplementary file 2 — SPIRIT figure. (PDF 311 kb) [file 13063_2017_1970_MOESM2_ESM.pdf]

**Figure 2** Schedule of enrolment, interventions, and assessments

| | **STUDY PERIOD** | | | |
|---|---|---|---|---|
| | Enrolment | Post-allocation | | |
| **TIMEPOINT** | *- t1* | Baseline *t1* | 6 months *t2* | 12 months *t3* |
| **ENROLMENT:** | | | | |
| Eligibility screen | X | X | | |
| Informed consent | | X | | |
| Allocation | | X | | |
| **INTERVENTIONS:** | | | | |
| (Usual care, Group-1) | | ◆—— | ———— | ——◆ |
| (Self-management, Group-2) | | ◆—— | ———— | ——◆ |
| (PCTM, Group-3) | | ◆—— | ———— | ——◆ |
| **ASSESSMENT:** | | | | |
| Systolic blood pressure | | X | X | X |
| Diastolic blood pressure | | X | X | X |
| BMI | | X | X | X |
| HbA1C | | X | X | X |
| Blood lipids | | X | X | X |
| Medication adherence | | X | | X |
